# Cortical dynamics of cold exposure and thermal recovery: Evidence from EEG‐based spatiotemporal analysis

**DOI:** 10.1113/EP093356

**Published:** 2026-01-22

**Authors:** Qing Zhang, Hetian Feng, Tian Liu, Jiaqi Zhou, Jingya Zheng, Junzhao Zhang, Li Ding, Jiachen Nie

**Affiliations:** ^1^ Beijing Advanced Innovation Center for Biomedical Engineering, Key Laboratory for Biomechanics and Mechanobiology of Ministry of Education, School of Biological Science and Medical Engineering Beihang University Beijing China; ^2^ Institute of Artificial Intelligence Beihang University Beijing China; ^3^ School of Emergency Management & Safety Engineering China University of Mining and Technology Beijing China

**Keywords:** cold stimulation, cortical response, EEG, thermal perception

## Abstract

Human thermal perception involves complex and dynamic interactions between peripheral input and central neural regulation. However, the spatial and temporal characteristics of brain responses to different cold exposure scenarios remain poorly understood. In this study, we combined traditional analysis with AI‐based anomaly detection to examine electroencephalographic (EEG) responses across five stages of cold exposure in 20 healthy participants, including baseline, cold exposure, wind stimulation, adaptation and recovery. Alpha‐band power analysis revealed 14 EEG channels with significant stage‐dependent differences, primarily located in the right hemisphere across frontal, central and parietal regions. Shapley additive explanations (SHAP)‐based feature importance scores further validated stage‐specific channels, identifying F8, T8 and CP6 for cold exposure, T7 for wind stimulation, T8 for adaptation, and F8 and CP6 for recovery. Time–frequency analysis revealed early spectral responses within 1 s for cold exposure and recovery, and within 2 s for wind stimulation, while AI anomaly detection estimated later latencies of 2.201∼2.735 s, highlighting the distinct sensitivities of each method. These results reveal right‐lateralized, stage‐specific brain activations, and demonstrate the complementary value of traditional and AI methods in decoding thermal responses.

## Abstract (highlights)

- **What is the central question of this study?**
  How does the human brain dynamically respond to varying stages of cold exposure?
- **What is the main finding and its importance?**
  There is a right‐lateralized, stage‐specific cortical activation pattern, characterized by distinct temporal latencies identified through both traditional and AI‐assisted analyses. These results provide novel evidence for a neurophysiology of cold perception and demonstrate the integrative value of combining conventional and machine‐learning approaches in thermal neuroscience.

## 1 INTRODUCTION

Thermoregulation enables humans to maintain a stable core temperature despite environmental challenges, relying on hypothalamic integration of peripheral thermal signals and coordinated autonomic responses such as vasoconstriction and thermogenesis (Gagge & Gonzalez, 2011; Morrison & Nakamura, 2019; Parsons, 2014; Werner, 2010). Cold exposure, particularly when combined with wind chill, strongly activates these regulatory pathways and evokes both behavioral and physiological defenses that minimize heat loss (Castellani & Young, 2016). Despite extensive physiological research, the neural mechanisms underlying human thermoregulation remain poorly understood (Morrison & Nakamura, 2019; Tan & Knight, 2018).

Electroencephalography (EEG) provides a non‐invasive approach to link cortical dynamics with thermal perception and regulation, extending beyond peripheral indicators such as skin temperature and heart rate (Frank et al., 1999; Nakamura et al., 2008). Previous studies using localized or steady‐state thermal stimuli have shown that EEG rhythms – particularly in the δ, θ and α bands – vary systematically with heating or cooling intensity (Mulders et al., 2020; Zhu et al., 2020). These findings suggest that cortical activity reflects thermal sensation and comfort (He et al., 2022; Yao et al., 2009), and such neural indices have been mainly applied to thermal comfort modelling in controlled indoor environments (Yao et al., 2008).

In contrast, real‐world cold exposure involves transient and dynamic environmental changes that pose greater challenges to thermoregulatory control. Systematic EEG investigations under full‐body, air‐based cold conditions remain scarce, largely due to methodological difficulties in maintaining signal quality during such exposures. Localized cold‐water immersion studies have revealed various cortical responses. Several studies have examined EEG responses during hand immersion in cold water, investigating EEG power spectra (Chang et al., 2005; Dowman et al., 2008) and topographical distributions (Shao et al., 2012; Wang et al., 2015) associated with different localized thermal stimuli. Significant increases in α and β power have been observed (Backonja et al., 1991; Tayeb *et al.*, 2022), along with enhanced δ and θ activity in the frontal regions, decreased α activity in the posterior parietal cortex, and increased β activity in the bilateral temporal regions (Chang et al., 2002a). Moreover, elevated θ and α power in the parietal lobe (Uragami & Osumi, 2023) and early cortical activations within 400–500 ms following strong cold stimuli in the prefrontal and midbrain regions have also been reported (Tayeb et al., 2022).

In addition to the above studies relying on water‐induced localized stimuli, most air‐based EEG–temperature studies have been conducted under mild and stable conditions (He et al., 2022; Kim et al., 2017; Son & Chun, 2018; Zhu et al., 2020), limiting their relevance to extreme cold environments. Research on harsher exposures has instead focused on peripheral or subjective indicators such as skin temperature, heart rate or thermal sensation (Liu et al., 2011; Wu et al., 2021; Zlatar et al., 2019), while the neural processing mechanisms remain largely unexplored. Moreover, although recent studies have introduced machine learning into thermal comfort classification and prediction (Maruyama et al., 2024), few have integrated such data‐driven approaches with physiological interpretability to probe the underlying neural mechanisms of cold stress.

Therefore, this study aimed to investigate the neural responses to different stages of cold exposure, with particular focus on low‐temperature exposure, cold temperature combined with wind stimulation, and the recovery phase. In addition to the transient responses associated with state transitions, sustained cortical activity after prolonged exposure was also analysed. EEG was used to quantify cortical dynamics across multiple regions, with an emphasis on identifying channel specificity and temporal evolution. Both traditional and AI‐assisted analytical methods were employed to characterize these patterns and validate findings across complementary approaches. Clarifying these mechanisms is critical for advancing the neurophysiological understanding of thermal regulation and for developing protective strategies in extreme environments, clinical hypothermia management, and occupational safety under thermal stress.

## 2 METHODS

### 2.1 Ethical approval

All experimental procedures were conducted in accordance with the *Declaration of Helsinki* and approved by the Institutional Ethics Committee of the School of Biological Science and Medical Engineering in Beihang University (Approval No. 20210149). Written informed consent was obtained from all participants prior to the experiments.

### 2.2 Subjects

A total of twenty male volunteers were recruited from the graduate schools of university, all of whom were mentally and physically healthy (Table 1). None of the subjects had cardiovascular or pulmonary impairments, nor had they been exposed to low‐temperature environments (below 0°C) for 2 months prior to the start of the experiments, or undergone cold acclimatization from an early age. All subjects participated voluntarily (experimental details are described below), with at least 3 h between any two consecutive experiments. Throughout the experimental period, subjects remained seated quietly on a chair with no work or entertainment activities permitted. They were instructed to obtain adequate sleep (>7 h per night, following their regular schedule). Consumption of caffeine and alcohol was prohibited the day prior to each experiment. All participants were fully briefed on the experimental procedures in advance and provided written informed consent.

**TABLE 1:** Physical details of the subjects.

Characteristic	Value
Height (cm)	172.0 (4.9)
Weight (kg)	71.5 (4.0)
Age (year)	23.3 (1.4)

Values are means (SD).

### 2.3 Experimental environments

**FIGURE 1:**
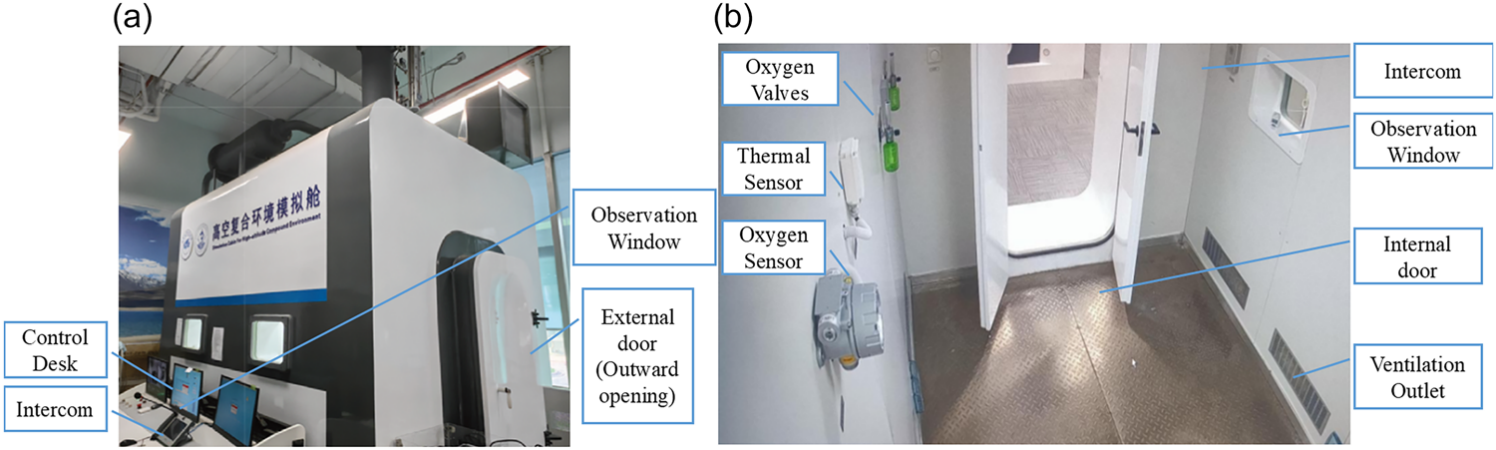
The sealed cabin for simulating low‐temperature environments showing the appearance (a) and internal structures (b) of the cabin.

Low‐temperature and high‐wind‐speed conditions were simulated in a fully sealed climate chamber measuring 3 × 2 × 2.7 m^3^, with a temperature range of −40 to 60°C (Figure 1). During the cold exposure periods, environmental conditions were systematically controlled according to predefined combinations of ambient temperature and wind speed. The tested thermal conditions are detailed in Table 2. To examine neural responses under different cold stress conditions, participants were randomly assigned to one of seven predefined environmental conditions that combined varying ambient temperatures and wind speeds. Specifically, two participants were assigned to the condition of −20°C and a wind speed of 7 m/s, while three participants were assigned to each of the remaining six conditions.

**TABLE 2:** Details of the experimental conditions.

Environmental parameters/conditions							
Atmospheric pressure (kPa)	101.3						
Ambient oxygen concentration (%)	21						
Ambient temperature (°C)	0	0	−5	−10	−15	−20	−20
Wind speed (m/s)	7	12	12	12	12	7	12
Number of subjects	3	3	3	3	3	2	3
Relative humidity (%)	40 ± 10						

The experimental conditions were designed to induce comparable levels of thermal stress, based on the working assumption that all combinations would be adequate to activate both peripheral and central nervous system responses. Therefore, it was hypothesized that *variations in temperature and wind speed would not result in significantly different neural response patterns across groups*. This assumption was empirically tested in subsequent statistical analyses to verify its validity.

### 2.4 Experimental procedures

#### 2.4.1 Preparation before test

Preparatory steps for the experiment were conducted under thermoneutral conditions (25°C, 30–40% relative humidity). All subjects were uniformed in a four‐layer clothing ensemble consisting of underwear, a wool–cotton blended thermal layer, a windproof layer and a thick coat, with a total thermal resistance of 3.535 clo (1 clo = 0.155 m^2^ K/W). An EEG electrode cap was placed on each participant, and the impedance of all electrodes was adjusted to below 5 kΩ.

#### 2.4.2 Experimental design

Each experimental session included four main phases: (i) a 5‐min baseline resting period at room temperature; (ii) a 10‐min cold exposure period in the climate chamber under conditions of no excessive airflow; (iii) a 40‐min cold exposure period with forced convection; and (iv) a 5‐min recovery period at room temperature. To capture transient neural responses associated with temperature‐related state transitions, EEG signals were recorded for 2 min at five key stages, as detailed in Table 3, including thermoneutral rest as baseline (NC), cold exposure (CE), cold wind stimulation (WE), habituation (HB), and post‐exposure recovery (PR).

**TABLE 3:** A single experimental session consisting of five stages designed to simulate thermal transitions.

Environmental stages	
Thermoneutral rest as baseline (NC)	5 min seated rest in the preparation room
Cold exposure (CE)	Inside the climate cabin set to exact temperatures (–20∼0°C)
Cold wind stimulation (WE)	10 min after entering the cabin, a fan was activated to deliver cold wind for 40 min (7 m/s or 12 m/s)
Habituation (HB)	During the 20‐min period after wind onset to capture neural responses
Post‐exposure recovery (PR)	After exiting the cabin, participants rested for 5 min in the thermoneutral environment

**FIGURE 2:**
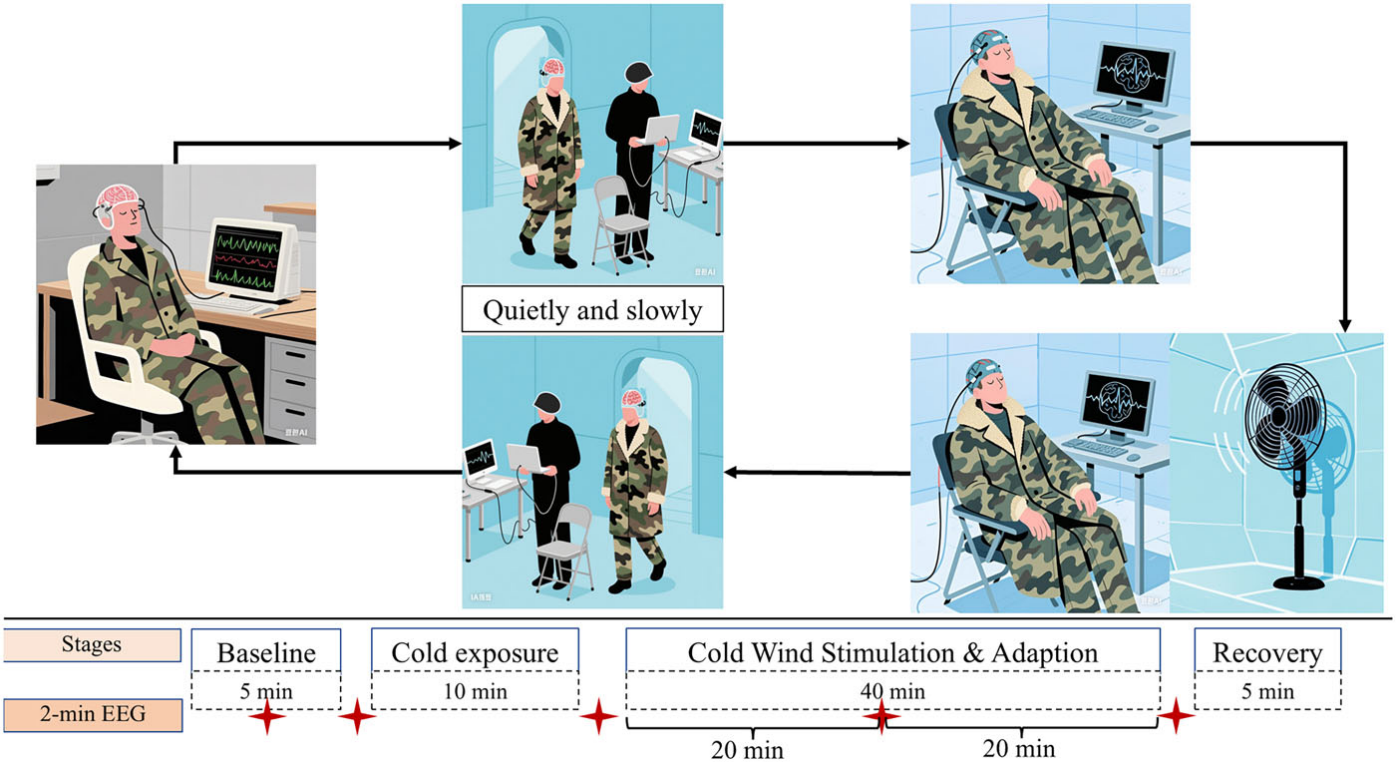
Overall workflow and key stages for capturing transient neural responses. The comic character was generated using the AI tool Doubao.

Transitions between experimental stages (i) to (ii) and (iii) to (iv) required participants to walk slowly and steadily between locations. To minimize motion‐induced artifacts, participants were instructed to perform all postural adjustments (e.g., standing up or sitting down) at a slow, consistent pace. EEG data were collected immediately during 2‐min windows at each stage transition, resulting in five distinct EEG epochs per participant. The experimental workflow is illustrated in Figure 2.

#### 2.4.3 Data acquisition

**FIGURE 3:**
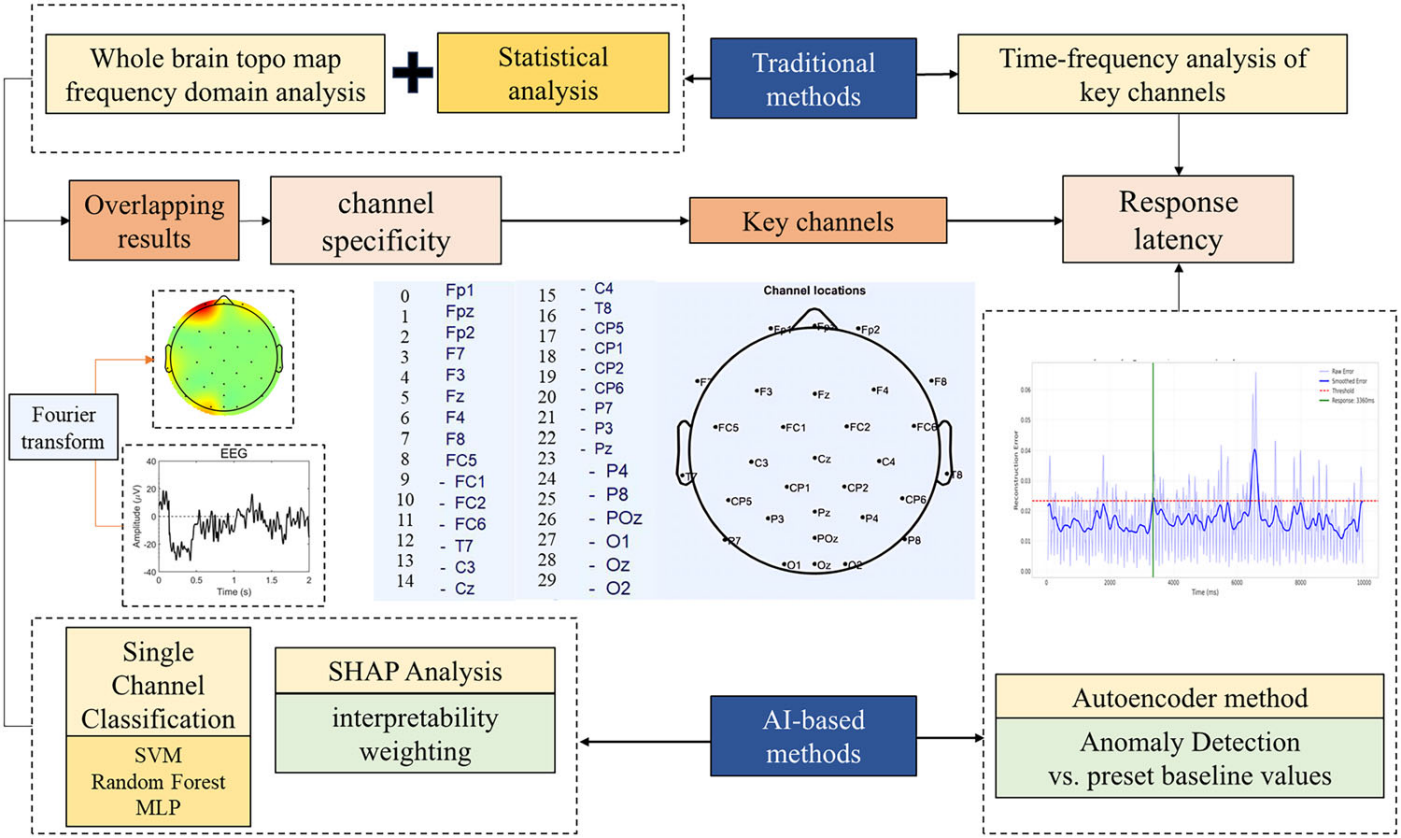
Overall analytical procedure in this study.

Cortical activity was assessed via EEG signals, which were acquired using a wired AgCl electrode cap (*Neuroscan*) with 32‐bit acquisition precision. Electrodes were positioned according to the International 10–20 System, encompassing 32 channels (Figure 3). Mastoid electrodes (M1 and M2) were used as references. The sampling frequency was set to 1024 Hz.

To validate the assumption outlined in Section 2.2, both skin temperature and subjective thermal sensation were measured. Local skin temperatures across multiple body segments were recorded using eight wireless sensors (iButton, DS 1922L; WDS Co., Ltd., Shanghai, China) with a measurement range of −40 to 80°C, with an accuracy of ±0.5°C and resolution of 0.1°C. Sensors were placed on the head, chest, back, upper arm, forearm, hand, thigh and calf. Weighted average skin temperature (WAST) was calculated using (Gagge & Nishi, 2011):

(1) $$\begin{array}{ccc} & & \mathrm{WAST}=0.070\times T_{\mathrm{head}}+0.175\times \left(T_{\mathrm{chest}}+T_{\mathrm{shoulder}}\right)+0.070\\ & & \times \;\left(T_{\mathrm{upper}\mathrm{arm}}+T_{\mathrm{forearm}}\right)+0.050\times T_{\mathrm{hand}}+0.190\times T_{\mathrm{thigh}}\\ & & +\;0.200\times T_{\mathrm{calf}} \end{array}$$

Subjective thermal sensation data were collected every 5 min using a questionnaire based on international standards (ASHRAE, 2017; ISO, 2005), with responses categorized into seven distinct levels for detailed analysis (Table 4).

**TABLE 4:** Subjective thermal sensation index.

Score	−3	−2	−1	0	1	2	3
Sensation	Very Cold	Cold	Cool	Comfortable	Warm	Hot	Very Hot

### 2.5 Sample size justification

This experimental design yielded 100 EEG segments (20 subjects × 5 distinct experimental stages), each with dimensions of 1024 Hz × 120 s. The sample size of 20 participants was determined via a priori power analysis using G*Power 3.1 (Faul et al., 2007; Xu et al., 2024). For a repeated‐measures within‐subject design with five conditions, a medium effect size (*f* = 0.25) (Cohen, 1988), alpha level of 0.05, and desired statistical power of 0.80, the analysis indicated a minimum required sample size of 18. The selected sample size of 20 ensures adequate power to detect moderate effects of thermal conditions on EEG metrics while accounting for potential data loss due to noise or artifacts. This sample size is consistent with previous EEG studies employing similar sensory paradigms and within‐subject comparisons in thermal neuroscience (Lv et al., 2017; Maruyama et al., 2024). The within‐subject design enhances statistical sensitivity, and each participant contributes multiple data points across controlled environmental stages, further strengthening the robustness of the results.

### 2.6 Data preprocessing

Raw 2‐min EEG data from each trial across all subjects were first preprocessed to mitigate the impact of background noise. Analyses were conducted using the EEGLAB toolbox in MATLAB 2022a, following a structured pipeline: (i) import raw data and verify electrode positions to ensure accurate channel localization; (ii) apply a 0.1–30 Hz bandpass filter to retain relevant EEG frequency components; (iii) manually identify and remove abnormal waveforms with irregular oscillatory patterns, and correct non‐functional channels via interpolation; (iv) eliminate physiological artifacts (e.g., eye movements, muscle activity) using independent component analysis (ICA).

### 2.7 Data analysis

This study focused on two dimensions of neural responses: channel specificity and temporal dynamics. The analytical framework was divided into traditional analysis and AI‐assisted analysis (Figure 3).

#### 2.7.1 Data set construction

The experiment included seven environmental conditions (varying ambient temperature and wind speed), resulting in 20 experimental datasets (one per participant). To determine whether these conditions could be pooled into a single dataset for subsequent analyses, we statistically evaluated the time‐domain feature of mean amplitude across all electrodes. Each 2‐min EEG segment was split into 120 consecutive 1‐s windows, and the average amplitude of each window was computed. For each experimental stage, statistical comparisons were performed across environmental conditions. The Shapiro–Wilk test and Levene's test were used to verify normality and homogeneity of variance, respectively. Welch's ANOVA was then employed to assess potential group differences.

#### 2.7.2 Traditional analysis of neural responses

As reviewed in the Introduction, brain regions associated with thermal responses are diverse, and findings remain inconsistent. Using the aforementioned 1‐s segmentation method, we computed the average amplitude across all 30 EEG channels (32 measured channels, excluding two reference electrodes) for each experimental stage. Normality and homogeneity of variance for global mean amplitude were confirmed via the Shapiro–Wilk and Levene's tests, respectively, while Mauchly's test was used to assess sphericity. ANOVA or Welch's ANOVA was applied to analyse global and individual channel mean amplitudes.

To characterize the spatial distribution of frequency‐specific responses to cold exposure, topographical EEG maps were generated based on the relative power of the δ (0–4 Hz), θ (4–7 Hz), α (8–12 Hz) and β (12–30 Hz) bands. The respective power spectra *P_x_* for each frequency band was calculated using:

(2) $$P_{x}=\frac{1}{n}\sum_{k=1}^{n}{\left|S_{k}\right|}^{2},\left(x=\delta ,\theta ,\alpha ,\beta \right)$$

where *n* represents the number of points of the discrete signal, and *S_k_* represents the amplitude of the discrete points of the time‐domain signal. Then, the relative power of the waveform in each frequency band was calculated as:

(3) $$P_{R}=\frac{P_{x}}{P_{\delta }+P_{\theta }+P_{\alpha }+P_{\beta }},\left(x=\delta ,\theta ,\alpha ,\beta \right)$$

In addition to topographical mapping, normality, homogeneity of variance and sphericity across the five stages were verified, followed by Welch's ANOVA for further statistical analysis. Hjorth complexity – an indicator of EEG spectral shape regularity – was computed to characterize signal complexity across stages. The Benjamini–Hochberg false discovery rate (FDR) correction was applied to account for multiple comparisons across channels and frequency bands.

Based on the stage‐sensitive channels identified above, time–frequency analysis was performed using the short‐time Fourier transform (STFT) to examine the temporal evolution of neural responses. This analysis was implemented via the spectrogram function in the Signal Processing Toolbox of MATLAB (MathWorks, Natick, MA, USA). A 500‐ms Hanning window was selected to balance temporal and frequency resolution. Analyses focused on 0–2 s post‐event onset, with power spectral density computed across 0–30 Hz.

#### 2.7.3 AI‐assisted analysis of neural responses

To further explore the condition specificity of EEG channels, machine learning classifiers were employed. For each channel, 5000 ms of EEG data were segmented into 100 overlapping 500‐ms windows. The time‐domain statistical feature (average amplitude) was extracted from each window and used as input for classification models tasked with discriminating among the seven cold exposure conditions. Three classifiers – multilayer perceptron (MLP), random forest (RF), and support vector machine (SVM) – were constructed for each channel, outputting multiclass classification results for the five experimental stages.

To avoid over‐reliance on raw classification accuracy, which may be limited for single‐channel inputs, the SHapley Additive exPlanations (SHAP) method (Lundberg & Lee, 2017) was used to quantify each channel's importance in model predictions. This approach enabled identification of channels specifically informative for discriminating individual stages, rather than those broadly influential across all conditions.

To complement traditional time–frequency methods, *autoencoders* – an unsupervised neural network class (Iqbal & Qureshi, 2022; Malhotra et al., 2015; Zhou & Paffenroth, 2017) – were employed to detect the precise onset of individual‐level neural responses. For each participant and condition, the first 10 s of EEG data post‐stimulus onset were segmented into non‐overlapping 50‐ms windows. Each participant‐specific autoencoder was trained exclusively on their baseline EEG data, capturing the unique distribution of neural activity in a resting, unstimulated state. Neural responses in the CE, WE and PR stages were defined as the earliest time point at which reconstruction error exceeded a predefined threshold.

## 3 RESULTS

This study examined the neural correlates of distinct cold exposure conditions and durations, with a particular focus on three types of thermal transitions: ambient temperature exposure, cold temperature combined with forced convection, and the subsequent recovery stage. Both transient and sustained EEG responses were analysed.

### 3.1 Data integration

The Shapiro–Wilk test confirmed that data distributions for each environment conformed to normality assumptions (for NC, CE, WE, HB and PR stages, *P* = 0.650, 0.646, 0.308, 0.443 and 0.289, respectively). Levene's test indicated that the homogeneity of variance assumption was violated across environments during HB and WE stage (for NC, CE and PR stages, *P* = 0.515, 0.437 and 0.580, respectively; while for HB and WE stage, *P* = 0.045 and 0.047, respectively). The results of Welch's ANOVA showed no significant differences in EEG responses across different environmental conditions (for NC, CE, WE, HB and PR stages, *P* = 0.850, 0.056, 0.068, 0.143 and 0.689, respectively), suggesting the pooling of all 20 participant datasets into a single analysis cohort was reasonable and feasible, thereby enhancing the statistical power and robustness of subsequent analyses.

**FIGURE 4:**
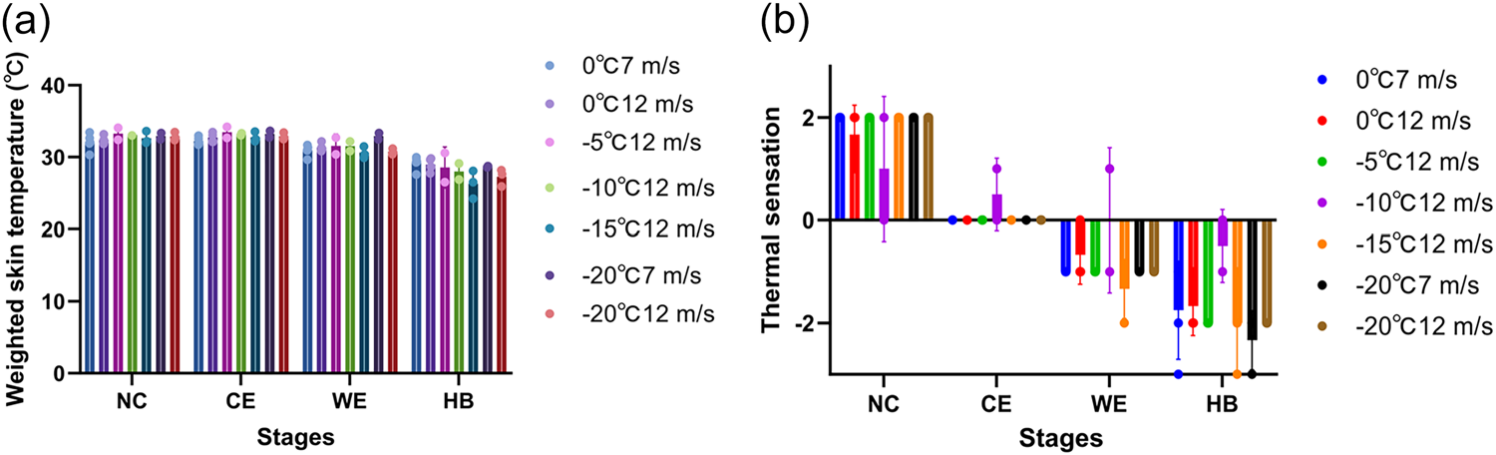
The results of weighted average skin temperature (a) and thermal sensation (b) at NC, CE, WE and HB stages under seven experimental conditions.

The results of skin temperatures and subjective thermal sensation could also validate the appropriateness of the pooling. As exhibited in Figure 4, consistent patterns emerged across the seven experimental conditions. At the CE stage, WAST did not show significant reductions compared with the NC stage, which is consistent with physiological expectations that short‐term cold exposure does not immediately lower body temperature due to thermoregulation. In contrast, thermal sensation significantly decreased at CE in almost all conditions (except −10°C/12 m/s), indicating a rapid perceptual response to cold stress. At the WE stage, WAST showed a slight decrease (except in −20°C, 7 m/s), while subjective cold sensation further intensified. Following 20 min of combined cold exposure and wind (HB stage), WAST exhibited significant reductions across all conditions, and thermal sensation further declined.

Occasional deviations may reflect individual variability rather than systematic effects. Notably, differences among conditions were not amplified by environmental severity. For instance, the most extreme condition (−20°C, 12 m/s) did not consistently yield the lowest skin temperatures or thermal sensation ratings, while the mildest condition (0°C, 7 m/s) was not always the highest. This pattern suggests that, within the short‐term exposure duration of this study, thermoregulatory mechanisms could maintain skin temperature within a compensable range and perceptual responses did not scale linearly with environmental parameters.

### 3.2 Channel‐specific analyses

#### 3.2.1 Time‐domain analysis of neural responses across stimulation stages

The assumptions of normality (Shapiro–Wilk test, *P* = 0.602), homogeneity of variance (Levene's test, *P* = 0.300) and sphericity (Mauchly's test, *P* = 0.182) were all satisfied. The ANOVA revealed no significant differences in global mean EEG amplitude across the five experimental stages (*P* = 0.178), suggesting an overall stable cortical excitability under varying cold exposure conditions. To explore the possibility of region‐specific neural modulation, we repeated the same statistical procedure for the 30 individual channel. This analysis identified significant stage‐related differences in mean amplitude at the Fp2, F3 and Cz electrodes (FDR‐corrected *P* = 0.017, 0.024 and 0.043, respectively) (Tables 5 and 6), indicating stage‐sensitive activity in the right prefrontal, left frontal and central cortical regions.

**TABLE 5:** Stage‐dependent variations in EEG mean amplitude: Descriptive results of channel‐specific analysis.

Condition	Fp2	F3	Cz
NC	−0.15 (1.76)	−0.90 (1.86)	−0.22 (0.94)
CE	5.60 (10.79)	2.22 (4.41)	4.33 (10.24)
WE	0.52 (5.55)	−0.52 (3.32)	−0.47 (3.21)
HB	−2.19 (7.49)	5.02 (9.04)	−1.09 (6.43)
PR	−0.63 (6.79)	−1.65 (11.36)	−1.20 (5.24)

Values are means (SD).

**TABLE 6:** Stage‐dependent variations in EEG mean amplitude: Statistical results of channel‐specific analysis.

	*F* (4, 76)	*P*	FDR‐corrected *P*	Partial η^2^
Fp2	4.045	0.00035	0.017	0.130
F3	3.473	0.00077	0.024	0.110
Cz	2.960	0.00162	0.043	0.087

#### 3.2.2 Frequency‐domain analysis of stage‐specific cortical activation

**FIGURE 5:**
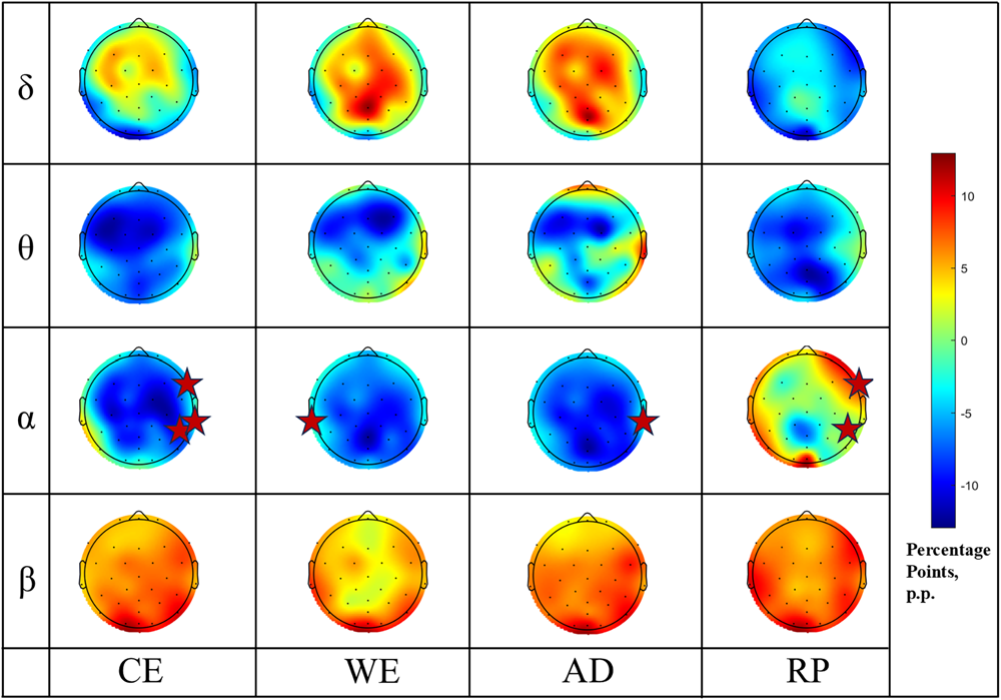
Topographic distribution of δ–α power changes across stages, with AI‐identified specific channels marked.

For each post‐stimulation stage, the relative power was baseline‐corrected by subtracting the corresponding pre‐stimulation (baseline) values, thereby highlighting thermal responses. These changes were visualized in topographic plots, and specific regions showing marked differences were summarized in a corresponding table (Figure 5, Tables 7 and 8). The statistical analysis revealed that only the α band exhibited significant stage‐dependent differences. Specifically, 14 channels showed significant changes in α power across the five stages in accordance with FDR‐corrected *P‐*values, including F8, FC5, FC2, FC6, Cz, C4, CP2, CP5, CP6, T7, T8, P4, POz and P7.

**TABLE 7:** Descriptive results of electrodes with significant changes in α power across five experimental stages.

Channels/conditions	NC	CE	WE	HB	PR
C4	0.45 (0.18)	0.31 (0.19)	0.23 (0.17)	0.35 (0.12)	0.31 (0.19)
T8	0.43 (0.20)	0.31 (0.20)	0.22 (0.15)	0.30 (0.13)	0.29 (0.19)
CP2	0.45 (0.19)	0.31 (0.21)	0.23 (0.17)	0.35 (0.15)	0.30 (0.21)
Poz	0.49 (0.19)	0.37 (0.23)	0.27 (0.14)	0.42 (0.20)	0.43 (0.21)
FC6	0.40 (0.18)	0.28 (0.19)	0.21 (0.16)	0.32 (0.13)	0.27 (0.16)
T7	0.37 (0.18)	0.27 (0.18)	0.19 (0.15)	0.29 (0.16)	0.23 (0.14)
Cz	0.40 (0.17)	0.27 (0.20)	0.22 (0.15)	0.34 (0.14)	0.29 (0.15)
P7	0.40 (0.20)	0.30 (0.21)	0.21 (0.12)	0.32 (0.16)	0.30 (0.18)
CP6	0.45 (0.20)	0.32 (0.21)	0.25 (0.17)	0.36 (0.14)	0.33 (0.21)
P4	0.46 (0.19)	0.34 (0.22)	0.26 (0.16)	0.37 (0.14)	0.36 (0.19)
FC2	0.37 (0.20)	0.27 (0.20)	0.20 (0.16)	0.34 (0.16)	0.26 (0.15)
CP5	0.28 (0.16)	0.20 (0.11)	0.15 (0.10)	0.23 (0.15)	0.18 (0.14)
F8	0.35 (0.17)	0.25 (0.17)	0.19 (0.15)	0.28 (0.12)	0.25 (0.15)
FC5	0.30 (0.16)	0.21 (0.14)	0.16 (0.13)	0.22 (0.15)	0.19 (0.14)

Values are means (SD).

**TABLE 8:** Statistical results of electrodes with significant changes in α power across five experimental stages.

	*F* (4, 76)	*P*	FDR‐corrected *P*	Partial η^2^
C4	5.845	0.00035	0.009	0.174
T8	5.371	0.00077	0.010	0.163
CP2	4.935	0.00162	0.014	0.153
Poz	4.533	0.00231	0.015	0.147
FC6	4.162	0.00308	0.016	0.132
T7	3.819	0.00438	0.019	0.121
Cz	3.503	0.00538	0.020	0.111
P7	3.327	0.00800	0.026	0.110
CP6	3.310	0.00935	0.027	0.090
P4	3.300	0.01077	0.028	0.087
FC2	3.287	0.01438	0.034	0.086
CP5	3.261	0.01800	0.039	0.085
F8	2.976	0.02050	0.041	0.085
FC5	2.939	0.02531	0.047	0.085

#### 3.2.3 Nonlinear dynamics of neural responses across stimulation stages

Among all channels, only FC2 showed a statistically significant difference in Hjorth complexity across the five stages (*F*(4,76) = 2.509, *P* = 0.025, FDR‐corrected *P* = 0.048, partial η^2^ = 0.074), pointing again to the right prefrontal cortex as a region of interest. This finding complements the frequency‐domain analysis and supports the presence of nonlinear dynamics in cortical adaptation to cold stress.

Moreover, the partial η^2^ values reported above (including in Tables 6 and 8), which ranged from 0.074 to 0.174 (corresponding to Cohen's *f* values of 0.28 to 0.46), indicate medium‐to‐large effect sizes. Therefore, the selection of *f* = 0.25 (a medium effect size) for our a priori power analysis using G*Power was conservative, further confirming the adequacy of the sample size in this study.

#### 3.2.4 Identification of stage‐specific channels using AI‐based classification models

**FIGURE 6:**
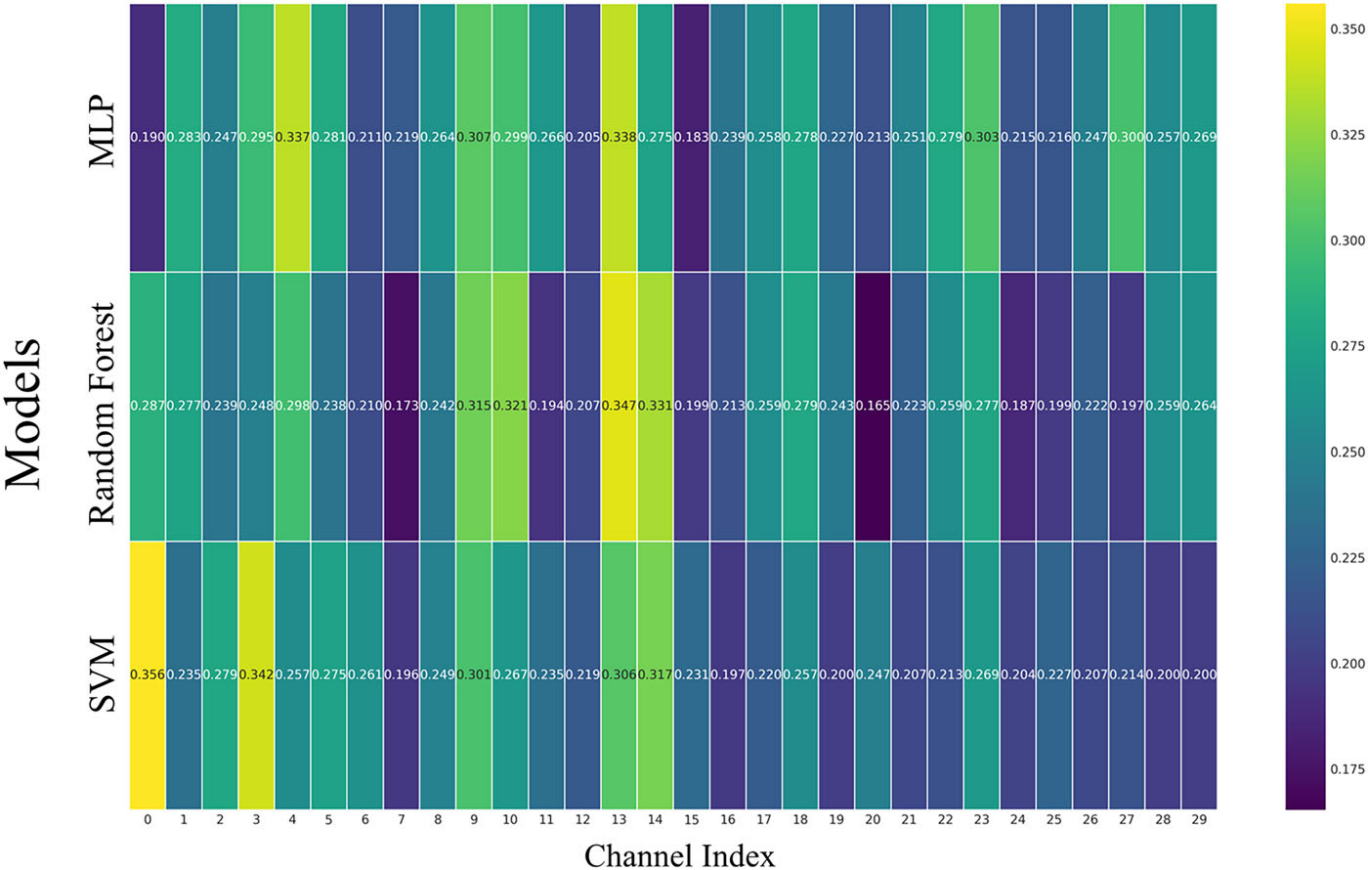
Classification accuracy of three machine learning models based on single‐channel EEG features.

For each channel, the classification accuracy of three models was recorded. However, none of the channels achieved high accuracy (usually, ≥70% accuracy was considered indicative of acceptable discrimination). Furthermore, no consistent high‐performing channels were identified across all three classifiers (Figure 6). These findings suggest that the EEG signals of a single channel do not contain sufficient information to robustly discriminate among all five stages.

**FIGURE 7:**
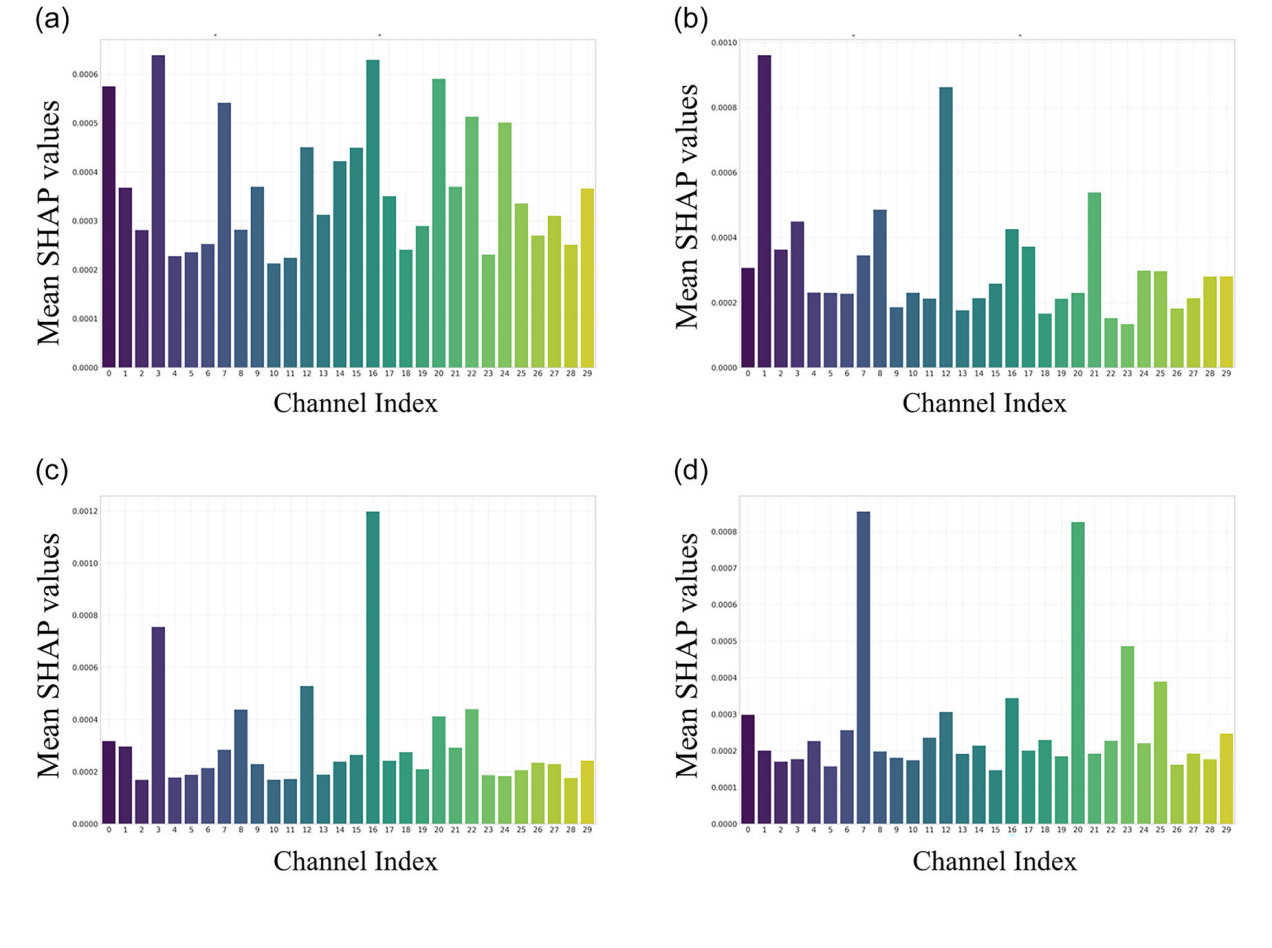
Stage‐specific important EEG channels identified using SHAP analysis. Mean SHAP values of each channel during CE (a), WE (b), HB (c) and PR (d).

To address the limitations of multiclass classification, a binary classification framework was developed, aiming to identify EEG channels that are particularly informative for specific stages. The results revealed the following channels associated with each condition, providing a refined map of stage‐specific cortical contributions under various conditions (Figure 7). In the CE stage, F7, T8, CP6, Fp1 and F8 showed the highest importance, suggesting early sensory processing and stress response involvement. In the WE stage, Fpz, T7 and P7 were most informative, likely reflecting wind‐specific somatosensory and parietal integration. In the HB stage, T8 and F7 emerged as dominant channels, possibly reflecting autonomic regulation and longer‐term cortical adjustment. In the PR stage, F8 and CP6 showed consistent high importance, indicating their role in post‐stress cortical reorganization. The channels have been highlighted with an asterisk in Figure 5.

### 3.3 Temporal dynamics of neural responses to cold stimuli

In the preceding sections, stage‐specific EEG channels were identified using both traditional statistical methods based on significance across time‐, frequency‐ and complexity‐domain features and AI‐driven SHAP modelling. Overlapping channels identified by both methods were considered high‐confidence targets for temporal analysis.

#### 3.3.1 Time–frequency analysis of identified stage‐specific channels

**FIGURE 8:**
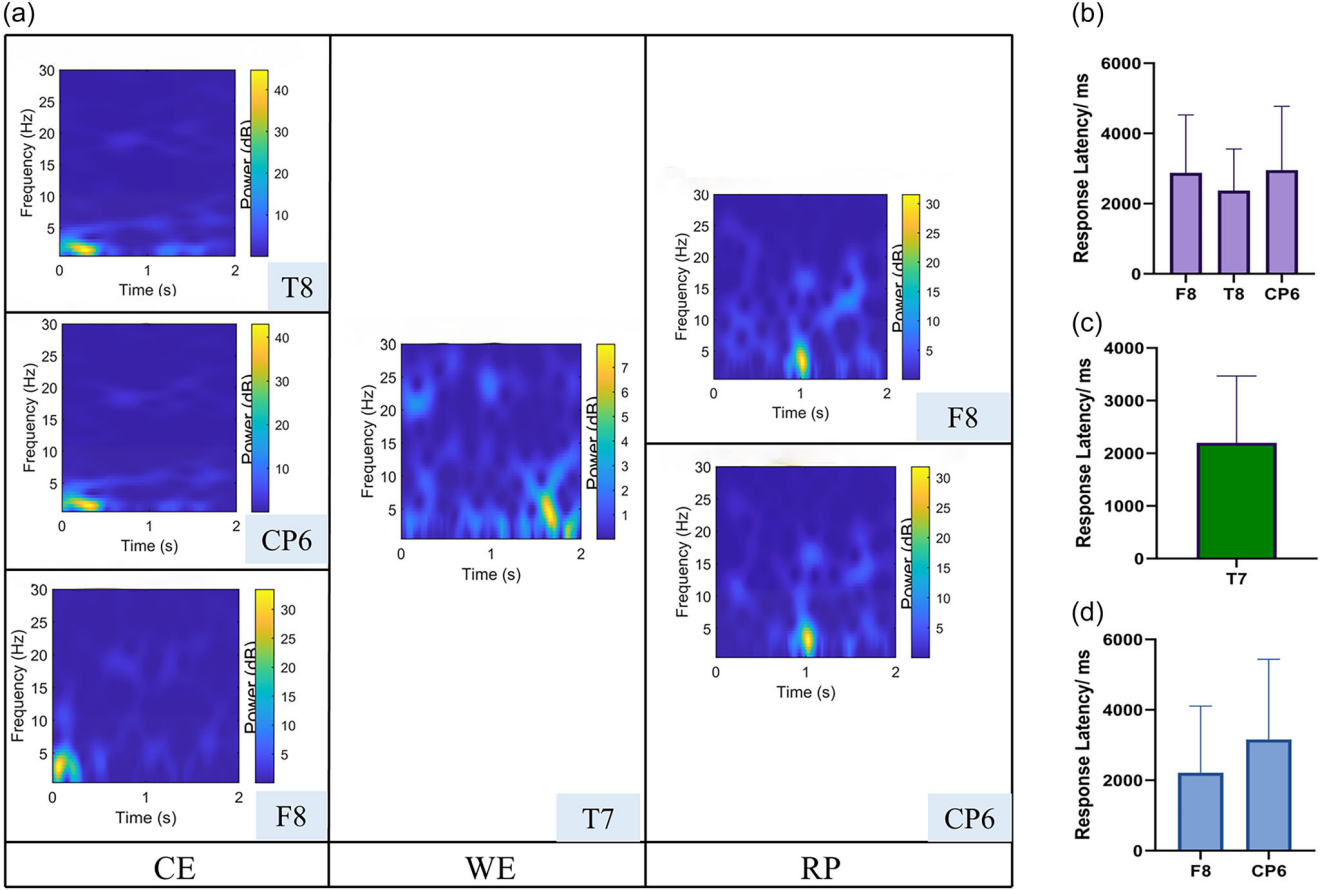
Temporal dynamics of cortical responses across three stages. (a) Latency of EEG responses derived from time–frequency analysis. (b–d) Latency estimated using anomaly detection for CE (b), WE (c), and PR (d) stages.

The analysis revealed distinct latencies for cortical activation depending on the type of stimulus (Figure 8a). Neural responses to initial cold exposure (CE stage) emerged within approximately 0.5 s, consistent with a rapid sensory processing mechanism, while responses to wind stimulation (WE stage), a more intense form of convective cold stress, showed a slightly delayed onset, occurring within 2 s. During the recovery (PR stage), cortical patterns gradually returned toward baseline levels, with observable shifts stabilizing within approximately 1 s, indicating a fast reestablishment of resting‐state dynamics. These findings suggest that different types of thermal perturbations engage cortical circuits at distinct temporal scales, which may correspond to the complexity and intensity of the thermal stimulus.

#### 3.3.2 Detection of neural response latency via AI‐based anomaly detection

The AI‐based anomaly detection approach was based on the hypothesis that EEG activity following cold exposure would deviate significantly from baseline patterns, marking the temporal onset of cortical activation. The reconstruction error was computed for each segment, yielding a temporal profile of neural deviation from baseline. For baseline data, reconstruction errors are minimal. However, when the model is presented with EEG data from the post‐stimulus period, the reconstruction error increases due to the presence of unfamiliar neural patterns. The results do not closely align with the time–frequency findings (Figure 8b–d). The mean response latency was estimated to be 2.735 ± 1.119 s for cold exposure (CE stage), 2.201 ± 1.268 s for cold wind stimulation (WE stage), and 2.688 ± 1.894 s for recovery (PR stage). These results provided an individualized estimation of the temporal neural responses, and showed that the brain responds more rapidly to dynamic, high‐intensity stimuli (e.g., wind) than to static cold exposure.

## 4 DISCUSSION

### 4.1 Channel‐specific neural responses: cortical activation characteristics under cold stimulation

This study integrated statistical analysis and AI‐based modelling to elucidate the spatial specificity of cortical regions activated across distinct stages of cold stimulation. Frequency‐domain analysis revealed significant differences in relative alpha power across the five cold stimulation stages in 14 channels, spanning multiple functional regions from the frontal and central areas to the right parietal and parieto‐occipital regions – with a predominance in the right hemisphere. These findings suggest and further support a potential lateralization pattern in cold perception (Kanosue et al., 2002).

Our results are highly consistent with previous research. For instance, Chang et al. (2002b) demonstrated that cold stimulation is associated with increased δ–θ–β power in the anterior temporal region and decreased α power in the posterior parietal region, reflecting attentional modulation and cortical regulatory mechanisms linked to nociceptive processing. In the present study, significant alpha power changes were observed in central and parietal channels (e.g., Cz, CP2, CP5, CP6, P4), which aligns closely with the established functional roles of these regions and reinforces the notion that cold stimuli trigger attention‐related modulation in the parietal cortex.

Stage‐specific channels identified via the SHAP method also correspond to known neurophysiological structures and prior findings. The postcentral gyrus of the parietal lobe functions as the primary somatosensory cortex, integrating incoming sensory information, while the precentral gyrus of the frontal lobe governs motor control and behavioural responses (Grefkes & Fink, 2005; Kandel et al., 2014; Yousry et al., 1997). During the initial cold exposure (CE) and wind stimulation (WE) stages, channels with high SHAP scores in the prefrontal, frontal and parietal regions indicate involvement of these areas in early sensory detection and prefrontal warning mechanisms. In the recovery (PR) stage, activation of channels F8 and CP6 may be associated with thermal pleasure responses to rewarming (Son & Chun, 2018). Additionally, extant literature indicates that intense stimuli such as cold wind can activate the insula, temporal cortex and limbic system – regions implicated in emotional regulation, pain encoding and autonomic control (Craig et al., 1996; Lindstedt et al., 2011). Our SHAP‐based identification of T8 as a key channel under prolonged cold and wind stimulation further underscores the central role of the temporal cortex in cold processing.

Notably, this study focused on overlapping channels identified by both traditional statistical analysis and AI models. While the classification accuracy of conventional AI approaches did not exceed the widely accepted 70% threshold, the supplementary application of the SHAP method provides complementary support for the physiological plausibility of these overlapping channels. Channel F8 (right prefrontal cortex) is associated with emotion regulation, risk assessment, proactive defence and sympathetic activation (Bechara et al., 2000; Critchley, 2005). Its consistent activation during both the CE and PR stages may reflect prefrontal modulation and vigilance adjustments during cold shock and thermal recovery. Channels T8 (right temporal) and T7 (left temporal) were identified in the CE, HB and WE stages, respectively, indicating that the temporal lobe contributes to the multidimensional integration of thermal and sensory information (Craig, 2003; Rolls, 2004). The repeated identification of T8 is particularly noteworthy, given its established roles in bodily state monitoring, pain memory and emotional processing (Chang et al., 2002b; Douglas et al., 2014), reflecting its potential adaptive engagement under prolonged cold exposure. Channel CP6, located at the centro‐parietal transition zone, is implicated in sensory–attentional integration and spatial localization. Its consistent activation during CE and PR suggests involvement in re‐establishing homeostatic cortical regulation during thermal transitions.

### 4.2 Temporal dynamics of neural responses to cold stimulation: latency, stage differences and cross‐method comparison

This study employed both time–frequency analysis and AI‐based anomaly detection to systematically evaluate the timing and dynamic evolution of neural responses across different cold stimulation states. While the two methods yielded differing estimates of response latency, this discrepancy reveals underlying differences in how the nervous system processes cold stimuli at multiple functional levels.

#### 4.2.1 Time–frequency analysis revealed earlier responses

Time–frequency analysis of channels identified by both statistical and AI‐based approaches showed that neural responses to CE, WE and PR emerged within 0.5 s, 2 s and approximately 1 s, respectively. These results indicate that cold stimulation triggers rapid and distinct changes in cortical spectral power – particularly in the alpha and theta bands – reflecting high perceptual sensitivity and fast cortical reactivity to thermal transitions. These findings align with previous research: Tayeb et al. (2022) reported that intense thermal stimuli can activate the prefrontal or parietal cortex within 400–500 ms. Our data further validate this observation and suggest that response timing and cortical localization vary by stimulus type. For example, wind stimulation may require more complex somatosensory integration, resulting in slightly delayed responses relative to static cold exposure.

#### 4.2.2 AI‐based anomaly detection reflected later response latencies

In contrast to time–frequency results, response latencies estimated via autoencoder‐based anomaly detection were relatively delayed: 2.735 s for CE, 2.201 s for WE and 2.688 s for PR, representing 440%, 10% and 160% longer latencies than those from time–frequency analysis. We hypothesize that this difference stems from the distinct definitions of ‘response’ adopted by the two methods. Time–frequency analysis is sensitive to rapid fluctuations in spectral power, enabling the detection of early local signal perturbations with millisecond precision. In contrast, the AI method establishes global baseline signal patterns and defines a response only when a significant deviation from this baseline is observed, resulting in more conservative timing estimates. In essence, the AI approach detects ‘systematic network‐level changes’, whereas traditional analysis captures ‘early local waveform dynamics’. Thus, the AI method is less responsive to the rapid dynamics of sensory processing and should be interpreted as providing a more conservative estimate of neural changes, reflecting slower cross‐regional network integration rather than immediate perceptual responses.

This methodological divergence offers a two‐tiered framework for understanding thermal sensory processing. On one hand, the brain initiates fast responses within hundreds of milliseconds to 1 s, representing the perceptual–sensory pathway. On the other hand, full‐scale cross‐regional network integration may require several seconds, representing the cognitive–regulatory pathway. Notably, AI‐based detection found that cold wind elicited faster responses than cold exposure (2.201 s < 2.735 s), aligning with physiological expectations that stronger stimuli elicit more rapid neural activation. Meanwhile, the delayed responses observed during the recovery stage may reflect homeostatic reorganization, thermal pleasure processing, or autonomic rebound mechanisms (Son & Chun, 2018). However, it should be noted that this dual‐process interpretation based on AI‐assisted analysis remains preliminary and exploratory, serving primarily to generate hypotheses for future verification rather than to establish definitive neurophysiological mechanisms.

### 4.3 Limitations and implications

In general, this study provides dual‐method characterization of the spatiotemporal cortical response patterns to staged cold stimulation. However, this study still has several limitations. Given the modest accuracy of the AI models and the limited sample size, the interpretation of AI‐derived patterns should be considered tentative and exploratory. These results provide potential insights into cortical regulation rather than conclusive evidence. First, the sample size was limited to 20 healthy young adult males, which limits the generalizability of the findings to other demographic groups (e.g., females, older adults, or individuals with impaired thermoregulation). Second, the study remains at a phenomenological level, aiming to identify stage‐specific cortical responses and response latencies under cold stimulation. While the findings offer new insight into the neural dynamics of thermal stress, they do not yet support direct application to real‐world systems such as wearable thermal feedback devices or intelligent environmental control. Additionally, as a surface‐based technique, EEG lacks depth resolution and may not fully capture subcortical structures involved in thermoregulatory control (e.g., hypothalamus). Future studies integrating multimodal imaging techniques – such as functional magnetic resonance imaging and positron emission tomography – are therefore needed to validate and extend these findings toward applied domains.

Although the present study demonstrates that AI‐based approaches can detect systematic changes in EEG during cold exposure, the current results should be interpreted as exploratory. The limited dataset constrains the training and optimization of the models, which partly explains the lower accuracy and longer response latencies compared to conventional analyses. Nevertheless, these shortcomings highlight the value of developing large‐scale EEG‐thermal physiology databases. With sufficient data diversity and volume, AI methods are expected to achieve higher accuracy and robustness through improved generalization across individuals. This is particularly important because traditional EEG analyses typically require subject‐specific baselines and are less effective for cross‐individual prediction. Besides, AI approaches may be particularly useful for capturing slower, integrative patterns of neural adaptation that complement conventional analyses of rapid sensory responses. Such scalability would represent a critical step toward clinical applications, from occupational safety monitoring to personalized environmental control systems.

### 4.4 Conclusion

This study presents an integrated investigation of the spatial and temporal features of cortical responses to cold exposure using EEG analysis, combining traditional statistical methods with AI‐based modelling. Our findings identify 14 α‐band channels, predominantly in the right frontal, central and parietal regions, as significantly modulated across five experimental stages. Key channels such as F8, T8 and CP6 were consistently highlighted by both statistical and AI approaches, underscoring their robust roles in cold processing. Moreover, time–frequency and AI‐based analyses revealed distinct response latencies, with early changes detected within 1∼2 s via traditional methods and delayed responses (2.201∼2.735 s) detected through AI anomaly modelling. These complementary findings suggest a two‐tiered cortical processing model for cold stimuli, involving rapid sensory detection and slower network‐level reorganization.

Methodologically, our integration of AI with physiological signal analysis offers a novel means to identify spatially and temporally specific brain features underpinning subjective thermal experiences. We expect that future work may refine the combination of traditional and AI approach to further quantify individual differences in thermal stimulation, model neurosensory processes, or guide the development of thermal comfort systems for environmental applications.

## AUTHOR CONTRIBUTIONS

Qing Zhang: Writing – original draft, Visualization, Methodology, Investigation, Conceptualization. Hetian Feng: Visualization, Methodology, Investigation. Tian Liu: Visualization, Formal analysis, Data curation. Jiaqi Zhou: Investigation, Data curation. Jingya Zheng: Investigation. Junzhao Zhang: Investigation. Li Ding & Jiachen Nie: Writing – review & editing, Data curation. All authors have read and approved the final version of this manuscript and agree to be accountable for all aspects of the work in ensuring that questions related to the accuracy or integrity of any part of the work are appropriately investigated and resolved. All persons designated as authors qualify for authorship, and all those who qualify for authorship are listed.

## CONFLICT OF INTEREST

None declared.

## FUNDING INFORMATION

None.

## Data Availability

All data supporting the findings of this study are available within the article. Any additional requests for information can be directed to, and will be fulfilled by, the corresponding author.

## References

[eph70153-bib-0001] American Society of Heating, Refrigerating and Air‐Conditioning Engineers . (2017). *ASHRAE Standard 55–2017: Thermal Environmental Conditions for Human Occupancy*.

[eph70153-bib-0002] Backonja, M. , Howland, E. W. , Wang, J. , Smith, J. , Salinsky, M. , & Cleeland, C. S. (1991). Tonic changes in alpha power during immersion of the hand in cold water. Electroencephalography and Clinical Neurophysiology, 79(3), 192–203. 1714810 10.1016/0013-4694(91)90137-s

[eph70153-bib-0003] Bechara, A. , Damasio, H. , & Damasio, A. R. (2000). Emotion, decision making and the orbitofrontal cortex (pp. 295–307). Oxford University Press. 10.1093/cercor/10.3.295 10731224

[eph70153-bib-0004] Bud Craig, A. D. (2003). A new view of pain as a homeostatic emotion. Trends in Neuroscience, 26(6), 303–307. 10.1016/s0166-2236(03)00123-1 12798599

[eph70153-bib-0005] Castellani, J. W. , & Young, A. J. (2016). Human physiological responses to cold exposure: Acute responses and acclimatization to prolonged exposure. Autonomic Neuroscience, 196, 63–74. 26924539 10.1016/j.autneu.2016.02.009

[eph70153-bib-0006] Chang, P. F. , Arendt‐Nielsen, L. , & Chen, A. C. (2005). Comparative cerebral responses to non‐painful warm vs. cold stimuli in man: EEG power spectra and coherence. International Journal of Psychophysiology, 55(1), 73–83. 15598518 10.1016/j.ijpsycho.2004.06.006

[eph70153-bib-0007] Chang, P. F. , Arendt‐Nielsen, L. , & Chen, A. C. N. (2002a). Dynamic changes and spatial correlation of EEG activities during cold pressor test in man. Brain Research Bulletin, 57(5), 667–675. 11927371 10.1016/s0361-9230(01)00763-8

[eph70153-bib-0008] Chang, P. F. , Arendt‐Nielsen, L. , & Chen, A. C. N. (2002b). Dynamic changes and spatial correlation of EEG activities during cold pressor test in man. Brain Research Bulletin, 57(5), 667–675. 11927371 10.1016/s0361-9230(01)00763-8

[eph70153-bib-0009] Cohen, J. (1988). Statistical power analysis for the behavioral sciences. (2nd ed.). Routledge. 10.4324/9780203771587

[eph70153-bib-0010] Craig, A. D. , Reiman, E. M. , Evans, A. , & Bushnell, M. C. (1996). Functional imaging of an illusion of pain. Nature, 384(6606), 258–260. 8918874 10.1038/384258a0

[eph70153-bib-0011] Critchley, H. (2005). Neural Mechanisms of Autonomic, Affective, and Cognitive Integration. The Journal Of Comparative Neurology, 493(1), 154–166. 16254997 10.1002/cne.20749

[eph70153-bib-0012] Douglas, P. K. , Pisani, M. , Reid, R. , Head, A. , Lau, E. , Mirakhor, E. , Bramen, J. , Gordon, B. , Anderson, A. , Kerr, W. T. , Cheong, C. , & Cohen, M. S. (2014). Method for simultaneous fMRI/EEG data collection during a focused attention suggestion for differential thermal sensation. Journal of Visualized Experiments, 5(83), e3298. 10.3791/3298 PMC4063545 24429915

[eph70153-bib-0013] Dowman, R. , Rissacher, D. , & Schuckers, S. (2008). EEG indices of tonic pain‐related activity in the somatosensory cortices. Clinical Neurophysiology, 119(5), 1201–1212. 18337168 10.1016/j.clinph.2008.01.019 PMC2676940

[eph70153-bib-0014] Faul, F. , Erdfelder, E. , Lang, A. , & Buchner, A. (2007). G*Power 3: A flexible statistical power analysis program for the social, behavioral, and biomedical sciences. Behavior Research Methods, 39(2), 175–191. 17695343 10.3758/bf03193146

[eph70153-bib-0015] Frank, S. M. , Raja, S. N. , Bulcao, C. F. , & Goldstein, D. S. (1999). Relative contribution of core and cutaneous temperatures to thermal comfort and autonomic responses in humans. Journal of Applied Physiology, 86(5), 1588–1593. 10233122 10.1152/jappl.1999.86.5.1588

[eph70153-bib-0016] Gagge, A. P. , & Gonzalez, R. R. (2011). Mechanisms of Heat Exchange: Biophysics and Physiology. Comprehensive Physiology, 1994(11S14), 45–84.

[eph70153-bib-0017] Gagge, A. P. , & Nishi, Y. (2011). Heat exchange between human skin surface and thermal environment. In Handbook of physiology: Environmental physiology (pp. 69–92).

[eph70153-bib-0018] Grefkes, C. , & Fink, G. R. (2005). The functional organization of the intraparietal sulcus in humans and monkeys. Journal of Anatomy, 207(1), 3–17. 16011542 10.1111/j.1469-7580.2005.00426.x PMC1571496

[eph70153-bib-0019] He, X. , Wu, M. , Li, H. , Liu, S. , Liu, B. , & Qi, H. (2022). Real‐time regulation of room temperature based on individual thermal sensation using an online brain‐computer interface. Indoor Air, 32(9), e13106. 36168224 10.1111/ina.13106

[eph70153-bib-0020] International Organization for Standardization . (2005). *ISO 7730: Ergonomics of the thermal environment—Analytical determination and interpretation of thermal comfort using calculation of the PMV and PPD indices and local thermal comfort criteria*.

[eph70153-bib-0021] Iqbal, T. , & Qureshi, S. (2022). Reconstruction probability‐based anomaly detection using variational auto‐encoders. International Journal of Computers and Applications, 45, 1–7.

[eph70153-bib-0022] Kandel, E. R. , Schwartz, J. H. , Jessell, T. M. , Siegelbaum, S. A. , Hudspeth, A. J. , & Mack, S. (2014). Principles of neural science. Fifth Edition. McGraw‐Hill Education.

[eph70153-bib-0023] Kanosue, K. , Sadato, N. , Okada, T. , Yoda, T. , Nakai, S. , Yoshida, K. , Hosono, T. , Nagashima, K. , Yagishita, T. , Inoue, O. , Kobayashi, K. , & Yonekura, Y. (2002). Brain activation during whole body cooling in humans studied with functional magnetic resonance imaging. Neuroscience Letters, 329(2), 157–160. 12165401 10.1016/s0304-3940(02)00621-3

[eph70153-bib-0024] Kim, M. , Chong, S. C. , Chun, C. , & Choi, Y. (2017). Effect of thermal sensation on emotional responses as measured through brain waves. Building and Environment, 118, 32–39.

[eph70153-bib-0025] Lindstedt, F. , Johansson, B. , Martinsen, S. , Kosek, E. , Fransson, P. , & Ingvar, M. (2011). Evidence for thalamic involvement in the thermal grill illusion: An FMRI study. Public Library of Science ONE, 6(11), e27075. 22096519 10.1371/journal.pone.0027075 PMC3214046

[eph70153-bib-0026] Liu, H. , Tan, Q. , Li, B. , Tan, M. & , & Ma, X. (2011). Impact of cold indoor thermal environmental conditions on human thermal response. Journal of Central South University Of Technology, 18(4), 1285–1292.

[eph70153-bib-0027] Lundberg, S. M. , & Lee, S. (2017). A unified approach to interpreting model predictions. In Proceedings of the 31st International Conference on Neural Information Processing Systems , pp. 4768–4777. Curran Associates Inc., Long Beach, California, USA.

[eph70153-bib-0028] Lv, B. , Su, C. , Yang, L. , & Wu, T. (2017). Effects of stimulus mode and ambient temperature on cerebral responses to local thermal stimulation: An EEG study. International Journal of Psychophysiology, 113, 17–22. 28082129 10.1016/j.ijpsycho.2017.01.003

[eph70153-bib-0029] Malhotra, P. , Vig, L. , Shroff, G. , & Agarwal, P. (2015). Long Short Term Memory Networks For Anomaly Detection In Time Series .

[eph70153-bib-0030] Maruyama, Y. , Nakamura, R. , Tsuji, S. , Xuan, Y. , Mizutani, K. , Okaze, T. , & Yoshimura, N. (2024). Classification of pleasantness of wind by electroencephalography. Public Library of Science ONE, 19(2), e0299036. 38412198 10.1371/journal.pone.0299036 PMC10898722

[eph70153-bib-0031] Morrison, S. F. , & Nakamura, K. (2019). Central Mechanisms for Thermoregulation. Annual Review of Physiology, 81(1), 285–308. 10.1146/annurev-physiol-020518-114546 30256726

[eph70153-bib-0032] Mulders, D. , de Bodt, C. , Lejeune, N. , Courtin, A. , Liberati, G. , Verleysen, M. , & Mouraux, A. (2020). Dynamics of the perception and EEG signals triggered by tonic warm and cool stimulation. Public Library of Science ONE, 15(4), e0231698. 32324752 10.1371/journal.pone.0231698 PMC7179871

[eph70153-bib-0033] Nakamura, M. , Yoda, T. , Crawshaw, L. I. , Yasuhara, S. , Saito, Y. , Kasuga, M. , Nagashima, K. , & Kanosue, K. (2008). Regional differences in temperature sensation and thermal comfort in humans. Journal of Applied Physiology, 105(6), 1897–1906. 18845785 10.1152/japplphysiol.90466.2008

[eph70153-bib-0034] Parsons, K. (2014). Human Thermal Environments: The Effects of Hot, Moderate, and Cold Environments on Human Health, Comfort, and Performance. Third Edition. 10.1201/b1675010.1201/b16750

[eph70153-bib-0035] Rolls, E. T. (2004). The functions of the orbitofrontal cortex. Brain Cognition, 55(1), 11–29. 15134840 10.1016/S0278-2626(03)00277-X

[eph70153-bib-0036] Shao, S. , Shen, K. , Yu, K. , Wilder‐Smith, E. P. , & Li, X. (2012). Frequency‐domain EEG source analysis for acute tonic cold pain perception. Clinical Neurophysiology, 123(10), 2042–2049. 22538122 10.1016/j.clinph.2012.02.084

[eph70153-bib-0037] Son, Y. J. , & Chun, C. (2018). Research on electroencephalogram to measure thermal pleasure in thermal alliesthesia in temperature step‐change environment. Indoor Air, 28(6), 916–923. 29989216 10.1111/ina.12491

[eph70153-bib-0038] Tan, C. L. , & Knight, Z. A. (2018). Regulation of Body Temperature by the Nervous System. Neuron, 98(1), 31–48. 29621489 10.1016/j.neuron.2018.02.022 PMC6034117

[eph70153-bib-0039] Tayeb, Z. , Dragomir, A. , Lee, J. H. , Abbasi, N. I. , Dean, E. , Bandla, A. , Bose, R. , Sundar, R. , Bezerianos, A. , Thakor, N. V. , & Cheng, G. (2022). Distinct spatio‐temporal and spectral brain patterns for different thermal stimuli perception. Scientific reports, 12(1), 919. 35042875 10.1038/s41598-022-04831-w PMC8766611

[eph70153-bib-0040] Uragami, S. , & Osumi, M. (2023). Cortical oscillatory changes during thermal grill illusion. Neuroreport, 34(4), 205–208. 36719830 10.1097/WNR.0000000000001874 PMC10516167

[eph70153-bib-0041] Wang, J. , Yi, M. , Zhang, C. , Bian, Z. , Wan, Y. , Chen, R. , & Li, X. (2015). Cortical activities of heat‐sensitization responses in suspended moxibustion: An EEG source analysis with sLORETA. Cognitive Neurodynamics, 9(6), 581–588. 26557928 10.1007/s11571-015-9349-x PMC4635395

[eph70153-bib-0042] Werner, J. (2010). System properties, feedback control and effector coordination of human temperature regulation. European Journal of Applied Physiology, 109(1), 13–25. 19787369 10.1007/s00421-009-1216-1

[eph70153-bib-0043] Wu, J. , Hu, Z. , Han, Z. , Gu, Y. , Yang, L. , & Sun, B. (2021). Human physiological responses of exposure to extremely cold environments. Journal of Thermal Biology, 98, 102933. 34016355 10.1016/j.jtherbio.2021.102933

[eph70153-bib-0044] Xu, Z. , Gao, F. , Fa, A. , Qu, W. , & Zhang, Z. (2024). Statistical power analysis and sample size planning for moderated mediation models. Behavior Research Methods, 56(6), 6130–6149. 38308148 10.3758/s13428-024-02342-2

[eph70153-bib-0045] Yao, Y. , Lian, Z. , Liu, W. , Jiang, C. , Liu, Y. , & Lu, H. (2009). Heart rate variation and electroencephalograph–the potential physiological factors for thermal comfort study. Indoor Air, 19(2), 93–101. 19348034 10.1111/j.1600-0668.2008.00565.x

[eph70153-bib-0046] Yao, Y. , Lian, Z. , Liu, W. , & Shen, Q. (2008). Experimental study on physiological responses and thermal comfort under various ambient temperatures. Physiology & Behavior, 93(1‐2), 310–321. 17936860 10.1016/j.physbeh.2007.09.012

[eph70153-bib-0047] Yousry, T. A. , Schmid, U. D. , Alkadhi, H. , Schmidt, D. , Peraud, A. , Buettner, A. , & Winkler, P. (1997). Localization of the motor hand area to a knob on the precentral gyrus. A new landmark. Brain: A Journal Of Neurology, 120(1), 141–157. 9055804 10.1093/brain/120.1.141

[eph70153-bib-0048] Zhou, C. , & Paffenroth, R. C. (2017). Anomaly Detection with Robust Deep Autoencoders. In Proceedings of the 23rd ACM SIGKDD International Conference on Knowledge Discovery and Data Mining , pp. 665–674. Association for Computing Machinery, Halifax, NS, Canada. 10.1145/3097983.309805210.1145/3097983.3098052

[eph70153-bib-0049] Zhu, M. , Liu, W. , & Wargocki, P. (2020). Changes in EEG signals during the cognitive activity at varying air temperature and relative humidity. Journal of Exposure Science & Environmental Epidemiology, 30(2), 285–298. 31235789 10.1038/s41370-019-0154-1

[eph70153-bib-0050] Zlatar, T. , Torees Costa, J. , Vaz, M. , & Baptista, J. (2019). Influence of severe cold thermal environment on core and skin temperatures: A systematic review. Work, 62, 337–352. 30829644 10.3233/WOR-192868

